# Exposure to malaria affects the regression of hepatosplenomegaly after treatment for *Schistosoma mansoni *infection in Kenyan children

**DOI:** 10.1186/1741-7015-2-36

**Published:** 2004-09-27

**Authors:** Mark Booth, Birgitte J Vennervald, Anthony E Butterworth, Henry C Kariuki, Clifford Amaganga, Gachuhi Kimani, Joseph K Mwatha, Amos Otedo, John H Ouma, David W Dunne

**Affiliations:** 1 Division of Microbiology and Parasitology, Department of Pathology, University of Cambridge, Tennis Court Road, Cambridge, CB2 1QP, UK; 2 Danish Bilharziasis Laboratory, Jægersborg Alle 1D, 2920 Charlottenlund, Denmark; 3 Biomedical Research and Training Institute, P.O.Box CY 1753, Causeway, Harare, Zimbabwe; 4 Division of Vector Borne Diseases, Ministry of Health, P. O Box 54840, Nairobi, Kenya; 5 Kakamega Provincial Hospital, P.O. Box 560, Kakamega, Kenya; 6 Kenya Medical Research Institute, Nairobi, Kenya; 7 Maseno University, Kisumu, Kenya

## Abstract

**Background:**

*Schistosoma mansoni *and malaria infections are often endemic in the same communities in sub-Saharan Africa, and both have pathological effects on the liver and the spleen. Hepatosplenomegaly associated with *S. mansoni *is exacerbated in children with relatively high exposure to malaria. Treatment with praziquantel reduces the degree of hepatosplenomegaly, but the condition does not completely resolve in some cases. The present analysis focused on the possibility that exposure to malaria infection may have limited the resolution of hepatosplenomegaly in a cohort of Kenyan schoolchildren.

**Methods:**

Ninety-six children aged 6–16, from one community in Makueni district, Kenya, were treated with praziquantel. At baseline, all children had hepatomegaly and most had splenomegaly. The source of *S. mansoni *infection, a river, was molluscicided regularly over the following three years to limit *S. mansoni *re-infection, whereas malaria exposure was uninterrupted. Hepatic and splenic enlargement was assessed annually outside the malaria transmission season.

**Results:**

Children living in an area of relatively high exposure to both infections presented with the largest spleens before treatment and at each follow-up. Spleens of firm consistency were associated with proximity to the river. The regression of hepatomegaly was also affected by location, being minimal in an area with relatively low *S. mansoni *exposure but high exposure to malaria, and maximal in an area with relatively low exposure to both infections.

**Conclusions:**

The outcome of treating cases of hepatosplenomegaly with praziquantel in this cohort of Kenyan children depended strongly on their level of exposure to malaria infection. Furthermore, a residual burden of hepatosplenic morbidity was observed, which was possibly attributable to the level of exposure to malaria. The results suggest that exposure to malaria infection may be a significant factor affecting the outcome of praziquantel treatment to reduce the level of hepatosplenic morbidity.

## Background

Hepatosplenomegaly is a widespread but neglected condition affecting many communities in sub-Saharan Africa where both *Schistosoma mansoni *and malaria infections are endemic. Severely enlarged and hard organs may be an indicator of increased portal pressure, as well as pointing to an increased risk of portal hypertension and its sequelae, including oesophageal varices and haematemesis [1]. Historically, hepatomegaly in sub-Saharan Africa has been associated with *S. mansoni *infection, and on a global scale up to 8.5 million individuals may be affected [2], with the prevalence of organomegaly generally highest in children [3]. Enlargement of spleens has often been associated with the level of malaria transmission [4,5]. One prevailing suggestion has been that hepatosplenomegaly amongst younger children is attributable to malaria infection, whereas older children have acquired some level of immunity to malaria and hence if they do have hepatosplenomegaly it is likely to be attributable to schistosomiasis [6]. However, chronic splenomegaly associated with portal hypertension has been reported in Kenyan hospitals amongst residents from areas where both *S. mansoni *and malaria infections are endemic [7,8], and it has long been suggested that the presence of both infections may confound attempts to quantify the impact of either one [9,10]. Recently, it was observed that IgG3 antibody responses to *P. falciparum *schizont antigen (Pfs Ag) were higher in Kenyan children with *S. mansoni *infection and hepatosplenomegaly compared with children with infection but no hepatosplenomegaly [11], further implicating exposure to malaria in childhood as a risk factor for severe hepatosplenic morbidity.

Given that malaria and schistosome infections are often endemic in the same communities, and are both targets of large-scale but independent, intervention programmes, it is important to understand how continued exposure to one parasite may affect the outcome of intervention against the other. The current longitudinal study evaluated praziquantel treatment on the regression of hepatosplenomegaly amongst children living in an area with endemic *S. mansoni *infection and seasonal malaria infection [12,13]. Before treatment, there was a strong correlation between *S. mansoni *egg count and the degree of both hepatomegaly and splenomegaly [12]. However, spatial analysis then revealed heterogeneity in exposure to both infections, and in particular there was an exacerbation of splenomegaly amongst children living in an area of relatively high exposure to both malaria and *S. mansoni *infections [14]. The prevalence of hepatomegaly and splenomegaly decreased slowly during the three-year follow-up period, but had not completely abated by the end of the study [13]. Here, we report that the level of exposure to malaria infection was likely to have been an important factor in limiting the resolution of hepatosplenomegaly in these children.

## Methods

### Study area

**Figure 1:**
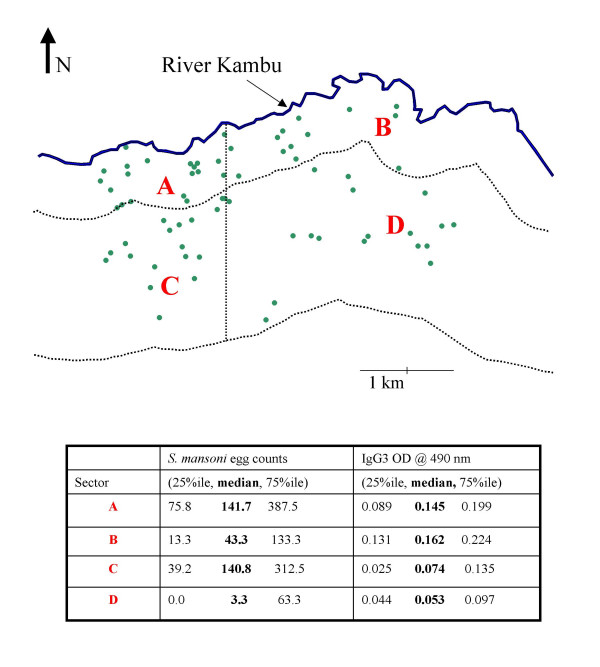
Map of the study area showing the course of the River Kambu, positions of cohort members households and the four sectors corresponding to areas of different exposure to *S. mansoni *and malaria infections. The accompanying table contains the median and 25–75%ile ranges for egg counts (e.p.g.) before treatment and anti-Pfs IgG3 OD responses for each sector.

Mbeetwani is situated in the district of Makueni, half way between Nairobi and Mombasa and approximately 10 km east of the main highway. The ethnic background of the population is predominantly Akamba. During the dry seasons, the river Kambu is the main source of water. Running from west to east, the river lies to the north of the community (Figure 1). Surface water is present at the western end during the dry season, whereas the eastern end is dry and water is retrieved by digging wells into the riverbed. River water is drawn for drinking, cooking and other domestic purposes. Washing of clothes and bathing takes place at numerous sites, and animals are taken to the river to drink.

### Clinical examination

The cohort and examination procedure are described in detail elsewhere [12]. Briefly, 96 children aged between 6 and 16 were selected for ultrasound detectable hepatomegaly after a community survey. They were examined before treatment with praziquantel at 40 mg/kg, and then examined again in the same month in the following three years. Treatment was given again two years into the study. All surveys took place in October of each year, towards the end of the dry season. A total of 67 children attended all examinations, whereas 71 children attended at least the baseline and third year follow-up surveys. Clinical examination consisted of liver and spleen palpation whilst children were in a supine position. Un-palpable spleens and livers were recorded as such, otherwise extensions in cm below the rib cage along the mid-clavicular line (MCL) and mid sternal line (MSL) were measured for the liver, and extension below the rib cage along the mid-clavicular and mid axillary lines (MAL) was recorded for the spleen. The firmness of each palpable organ was recorded as either soft, firm, or hard, as described previously [12]. An accompanying ultrasound examination was conducted according to World Health Organisation guidelines [15] to test for the presence of periportal fibrosis. Both the clinicians and the ultrasonographers were blinded as to the location of children's domiciles and previous examination results, and the same team performed the examinations throughout the study period.

### Mapping

Details of the mapping procedure are given elsewhere [14]. Briefly, the longitude and latitude of each house were recorded using a Magellan GPS 315 receiver. The course of the river Kambu was determined by taking readings approximately every 100 metres along a ten kilometre stretch that extended beyond the borders of the study area. The approximately rectangular study area was divided into 4 sectors with similar numbers of houses by bisecting the north-south and east-west transects at 4 km east of the western boundary and 1 km south of the river.

### Assays

Since the surveys were undertaken during the dry season, parasitaemia was not expected to provide a good enough estimate of exposure. Therefore, anti-Pfs IgG3 responses were used as a proxy measure of recent exposure [16], measured by ELISA as described previously [17].

### Parasitology

For the quantification of *S. mansoni *eggs, each child provided 3 stool samples before the baseline survey in October 1999. Two 50 mg Kato slides were prepared from each stool sample using the Kato-Katz procedure [18]. All slides were prepared and examined by the same team. Finger-prick blood samples were taken on the same day that clinical and ultrasound examinations were performed, for detection of malaria parasitaemia.

### Mollusciciding

Regular mollusciciding of the river Kambu was undertaken with Bayluscide ^® ^(Bayer CropScience AG, Alfred-Nobel-Str 50, D40789 Monheim am Rhein, Germany). Area-wide application and focal spraying using backpacks was undertaken twice yearly to coincide with periods when snail populations were reduced due to environmental conditions. Snail sampling activities at 9 sites on a monthly basis were undertaken to confirm that snail numbers were kept at very low levels (HC Kariuki, unpublished observations).

### Treatment and ethical considerations

The objectives of the study were carefully explained to the local community including the parents and teachers before the start of any activities. Informed assent was obtained from the parents or guardians of the children. After the clinical and ultrasound examination in October 1999, all children were treated with a single dose of praziquantel (Distocide^®^, Shin Poon Pharmaceuticals, Seoul, Republic of Korea), at 40 mg/kg body weight. The children were again treated in 2001 and 2002 under the same regimen. No other source of treatment was available locally during this period. Tablets were administered together with a piece of bread and a soft drink in order to minimise gastrointestinal side effects. The study was approved by the Kenya Medical Research Institute Ethical Review Committee.

### Analysis

Baseline data for each parameter were compared with 3 year follow up data amongst 71 children who attended the first and last surveys to estimate the number of children within each sector who presented with a lower degree of organomegaly at the end of the study. Repeated-measures ANOVA of each organ enlargement parameter was also undertaken for the 67 children who attended all surveys, with age, sex and sector of residence as fixed factors. Since ANOVA does not indicate the direction of any change, a *post-hoc *analysis was undertaken within each sector to determine whether there was an overall increase or decrease in the extent of organomegaly. This was achieved by comparing baseline enlargement and enlargement after 3 years follow up for each parameter, using the Wilcoxon Signed Ranks test. All analyses were conducted in SPSS v11 (SPSS inc, 2001, Chicago).

## Results

### Geographical distribution of exposure

Figure 1 illustrates the geographical distribution of houses within the study area, as well as the course of the river Kambu. A total of 70 houses were geo-referenced. Also given are the median and 25–75%ile range of egg counts and anti-Pfs IgG3 responses within each sector before the intervention. Details of the analysis of *S. mansoni *egg counts and IgG3 responses within each sector are given elsewhere [14]. Briefly, there was a significant clustering of high egg counts in children living at the western end of the study area (Sector A and Sector C). Significant clustering of the highest anti-Pfs IgG3 responses was observed amongst children living in the two northern sectors (Sector A and Sector B). Thus, sector A was an area of relatively high exposure to both infections, sector B was an area of relatively high exposure to malaria only, sector C was an area of relatively high exposure to *S. mansoni *only, and sector D was an area of low exposure to both infections.

### Temporal changes in organ consistency by sector

**Figure 2:**
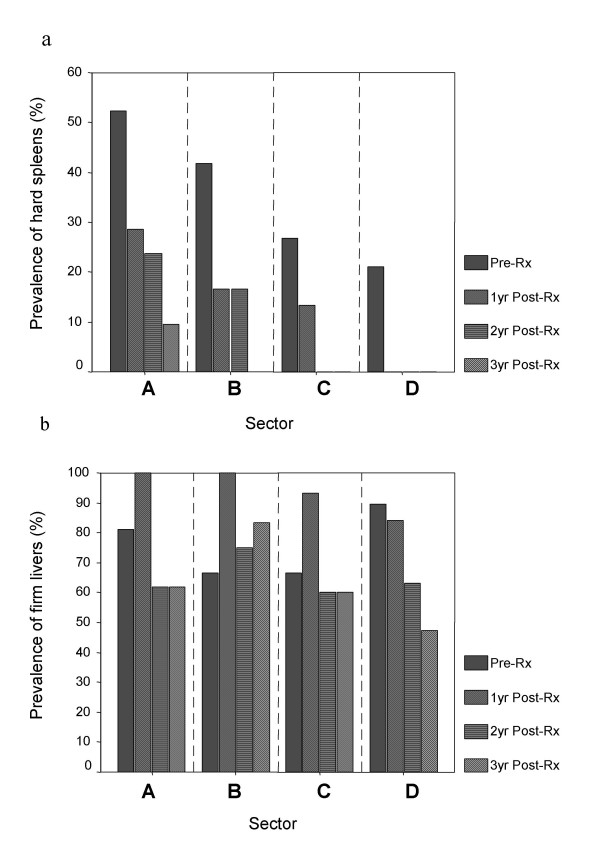
Bar charts depicting temporal (by year) and spatial (by sector) variation in the prevalence of hard spleens (Figure 2a) and firm livers (Figure 2b). Estimates are from the baseline survey and surveys conducted up to three years after first treatment (Rx) with praziquantel.

Significant variation in the prevalence of hard spleens over time was observed within each sector (Table 1), attributable to an overall decline in the number of children with hard and enlarged spleens (Figure 2a). The prevalence of hardened spleens was highest at all time points in the sector with relatively high anti-Pfs IgG3 responses and high egg counts (Sector A). By two years post-treatment, no child resident more than one kilometre from the river presented with a hard spleen.

The prevalence of firm livers in all sectors varied over time, except in sector B (Table 1). In all other sectors there was a decrease in the prevalence of firm livers over the course of the follow-up period (Figure 2b). At the end of the study, the lowest prevalence of firm livers was observed in children from sector D.

### Temporal changes in organomegaly by sector

**Figure 3:**
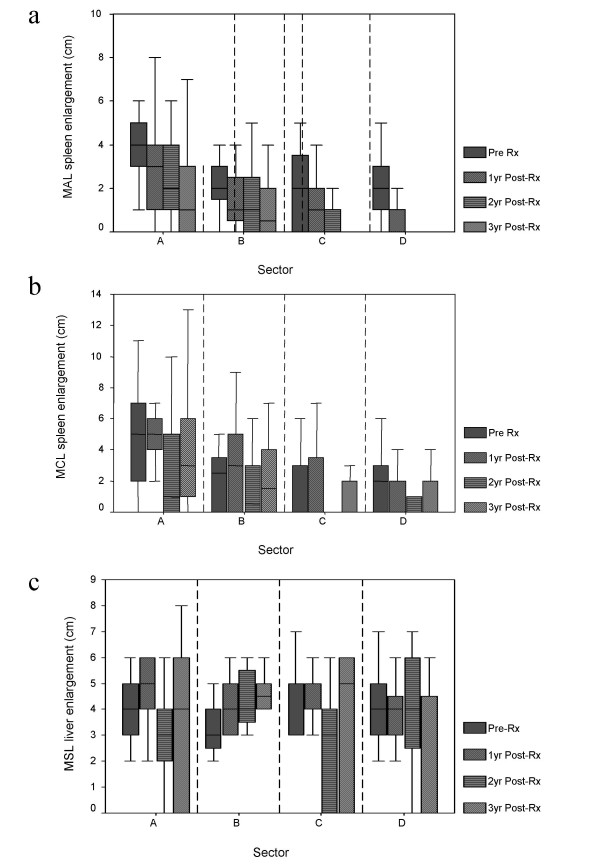
Box and whisker plots depicting temporal (by year) and spatial (by sector) variation in spleen enlargement along the mid axillary line (Figure 3a) and the mid clavicular line (Figure 3b), as well as liver enlargement along the mid sternal line (Figure 3c). The horizontal line within each box represents the median; the lower and upper bounds of the box correspond to the 25- and 75%iles; the whiskers correspond to the range of non-outlying data. Measurements are from the baseline survey and surveys conducted up to three years after first treatment (Rx) with praziquantel.

Significant variation in the degree of MAL splenomegaly was observed in each sector over the three-year follow-up (F = 20.7, p < 0.001). Figure 3 illustrates a decline in the degree of MAL splenomegaly within each sector, and this was confirmed by *post-hoc *Wilcoxon Rank analysis (Table 2). The rate of regression was affected independently neither by sector, age nor sex, and children from sector A presented with the largest spleens at each time point. At the third year post treatment follow-up, the fraction of each group presenting with no MAL splenomegaly in each sector was 10/24 (Sector A), 6/14 (Sector B), 13/15 (Sector C), and 16/20 (Sector D). MCL splenomegaly also varied significantly over time (Figure 3b, F = 14.1, p < 0.001), but the extent of variation was affected by sector (F = 2.191, p = 0.024). *Post-hoc *Wilcoxon rank analysis revealed that there was a significant decrease in the extent of MCL splenomegaly only in sector D (Table 2).

MSL liver enlargement varied significantly over time (F = 3.78, p = 0.012), with the variation being affected by sector (F = 2.57, p = 0.008). There was no consistent decline in MSL hepatomegaly over time within any one sector (Figure 3c). Although a fraction of the children in each sector presented with reduced hepatomegaly at the third year follow-up, Wilcoxon Rank analysis of the direction of change within each sector confirmed that an overall significant decrease in the extent of MSL hepatomegaly between the baseline survey and the third year follow-up occurred only amongst children from sector D (Table 2). Children from sector B presented with the least improvement in MSL hepatomegaly, with 10/12 exhibiting no change or an increase in this parameter when baseline data were compared with data from the third year follow-up. MCL hepatomegaly values could not be used in the repeated measures analysis due to a lack of normal variation in the data. Wilcoxon rank analysis within sectors revealed no significant change in this measurement between baseline and the third year follow-up in any sector (Table 2).

### Ultrasound results

There was no evidence of periportal fibrosis in any of the children at any time point.

## Discussion

Many clinical surveys of sub-Saharan communities have reported that organomegaly of the spleen or liver is a very common condition, but attribution to a specific aetiology has always been problematic. Distinguishing the contribution of malaria from that of schistosome infections is particularly complex since they are often endemic in the same communities. However, it is well established that exposure to both *S. mansoni *and malaria varies over small areas [19-21], and the potential therefore exists to compare morbidity in areas of overlapping exposure with areas where infections of one species are more prevalent. Here, we have exploited observations concerning micro-geographical variation in the distribution of each infection to demonstrate how the benefits of treatment with praziquantel on hepatosplenomegaly may be affected by local heterogeneity in malaria exposure. Our results further implicate both parasites as aetiological agents of chronic hepatosplenomegaly including firmness of the organs in school-aged children, and have strong implications not only for estimating the burden of *S. mansoni *and malaria, but also for estimating the outcome of interventions.

The generation of these results was facilitated by several important features of the study design. The intermittent mollusciciding of the river Kambu was particularly important, since it reduced the potential for morbidity to rebound as a result of re-infection, as has been seen elsewhere in studies of both *S. mansoni *and *S. haematobium *[22]. The timing of the follow-up surveys outside the malaria transmission season was another important component, since it allowed an assessment of the clinical situation without the confounding effects of transient morbidity associated with acute malaria. Without these particular features, it is unlikely that we could have come to any conclusions about the effects of chronic exposure to malaria infection on the regression of hepatosplenomegaly. Another important component of the study was the mapping of both house co-ordinates and the local river. By doing so, we were able to identify spatial clustering of relatively high *S. mansoni *egg counts at the western end of the study area. Such clustering is likely related to variation in the amount of surface water along the river, and hence variation in transmission potential due to the strong relationship between snail abundance and water level observed during long term studies of transmission in this area [23]. The clustering of anti-Pfs IgG3 responses to schizont antigen along a tract parallel to the course of the river suggests a sharp decrease in transmission further from the river [14].

Although we observed several significant changes following praziquantel treatment of the cohort, it is important to note that this was a retrospective analysis, which carries a few limitations. The observations made in this study were based on a small, case-only cohort, and therefore do not represent the outcome of an intervention programme involving mass treatment of a population. As this was a retrospective analysis, no control was possible concerning treatment for malaria during the follow-up period. The population had access to antimalarial drugs at local shops, but so far as we are aware there was no systematic intervention against malaria infection during the follow-up period. The results therefore encompass the effects of background treatment for, as well as exposure to, malaria infections.

Reliability of the clinical measurements is an important factor, especially given the size of the cohort. We have assessed this procedure elsewhere and it has been found to be satisfactory (unpublished observations). The involvement of three or four clinicians at each examination also reduced the degree of imprecision in the organomegaly measurements, and previous analysis has demonstrated that interpretable changes in the measurements occurred within this cohort [13].

The major result from this analysis is that the outcome of treatment with praziquantel, combined with very limited *S. mansoni *re-infection, depended strongly on where members of the cohort were resident. Previously, we demonstrated that children within the same cohort that had relatively high-level exposure to both *S. mansoni *and malaria presented with significantly larger spleens before treatment than children highly exposed to either parasite alone [14]. This contrasted with an observation on splenomegaly in school children exposed to malaria only in Ghana, where splenomegaly was observed to decline with distance from mosquito breeding sites [5]. Here, we report that children with relatively large spleens at baseline and living in the area with high levels of exposure to malaria still had the largest spleens 3 years after treatment, despite an overall reduction in their organomegaly.

Hardness of enlarged spleens at baseline was most commonly observed amongst children living close to the river, irrespective of their level of pre-treatment intensity of *S. mansoni *infection [14]. In the absence of follow-up data, the inference may have been that the hard consistency of enlarged spleens was primarily attributable to chronic malaria exposure. However, during the follow-up period, we observed a gradual and monotonic decrease in the prevalence of hard spleens that suggests the removal of *S. mansoni *infection was the trigger for the improvement [13]. One possible explanation is that the hardening of the spleens was due to a synergistic effect of co-infection. Hyperplasia and congestion in the spleen associated with relatively high levels of exposure to malaria may have been sufficient to cause chronic enlargement of the spleen. Hardening and further enlargement may then have occurred as a secondary effect of congestion in the liver attributable to schistosome infection, leading to increased portal pressure and dilation of the splenic vasculature.

Three years after treatment with praziquantel, and with very limited re-infection by *S. mansoni *[12], the prevalence of palpable spleens had diminished considerably. At all time points, the prevalence of hard spleens and the degree of splenomegaly were highest amongst children living in the sector with highest egg counts before intervention and highest anti-Pfs IgG3 responses. By removing *S. mansoni *infection and limiting re-infection, we may have abrogated any synergistic effects of co-infection on the spleen, and thereby uncovered the residual burden of chronic exposure to malaria. Importantly, the rate of regression of MAL splenomegaly was not affected significantly by location, which suggests that the effects of praziquantel treatment on splenomegaly attributable to *S. mansoni *infection are not dependent on the level of exposure to either malaria or *S. mansoni*.

The effects of praziquantel treatment on hepatomegaly in this cohort were more subtle. There was gradual decline in the prevalence of hepatomegaly after praziquantel treatment [13] indicating that the removal of *S. mansoni *infection was a critical factor for improvement. However, upon analysing hepatomegaly data across sectors, it emerged that children from the sector with relatively high anti-Pfs IgG3 responses, but relatively low *S. mansoni *egg counts, had the lowest rate of regression. A possible explanation of this observation is that an aetiological agent other than *S. mansoni *was responsible for the observed hepatomegaly. A likely candidate is malaria infection. Enlargement of the liver in acute malaria infection is temporary and recedes rapidly after treatment [24]; however, studies of young children in Gambia have shown that repeated infection with malaria, perhaps when combined with other unidentified factors, can lead to the development of chronically enlarged livers [25,26]. Our observations suggest that even if school-aged children are examined outside the malaria transmission season, they may still be affected by hepatomegaly attributable to malariainfection.

Children from the sector with relatively low exposure to both infections experienced a significant decrease in liver enlargement along the mid sternal line when baseline data were compared with data from the third year follow-up. It is possible that hepatomegaly in these children was attributable to neither *S. mansoni *nor malaria infection. Alternatively, because they were relatively lightly infected with *S. mansoni*, and experienced relatively low exposure to malaria infection, it is possible that they were more likely to regress in terms of hepatomegaly within the follow-up period.

## Conclusions

In conclusion, our observations lend further support to the hypothesis that severity of hepatosplenomegaly in Kenyan school-aged children is related to their degree of exposure to both *S. mansoni *and malaria infections. Specifically, although the degree of hepatomegaly or splenomegaly may be correlated with *S. mansoni *infection, there is likely to be further exacerbation of the condition if a child is concurrently exposed to malaria. We have now observed that the apparent benefits of treating a case of hepatosplenomegaly with praziquantel may be reduced if a child lives in an area of relatively high exposure. We have therefore confirmed the long-standing, but untested, hypothesis that co-infections of *S. mansoni *and malaria may obscure the clinical evaluation associated with infection by either species. Our results also introduce the necessity of considering the level of exposure to malaria when evaluating the clinical outcome of praziquantel treatment for *S. mansoni *infection.

## Competing interests

The author(s) declare that they have no competing interests.

## Authors' contributions

MB conceived of, and conducted, the analysis, and drafted the manuscript. BJV and AEB performed clinical examinations and participated in the design of the study. CA and AO conducted clinical examinations. CHK, GK, JM, JHO and EM participated in the planning and execution of field activities. DWD participated in the design of the study and in fieldwork.

## Pre-publication history

The pre-publication history for this paper can be accessed here:



## Figures and Tables

**Table 1:** Results of testing for variation in the prevalence of hard spleens and firm livers over time within each sector. *Sector *is described in the legend to table 1. *Q *– Cochrans Q statistic, *N *– number of individuals with relevant data from all time points, *p *– level of significance.

	Hard spleens			Firm Livers	
Sector	*N*	*Q*	*p*	*Q*	*p*
*A*	21	14.0	0.003	13.2	0.004
*B*	12	9.0	0.029	5.0	0.172
*C*	15	8.3	0.041	7.8	0.049
*D*	19	12	0.007	10.7	0.013

**Table 2:** Improvement in hepatosplenomegaly stratified by level of exposure to malaria and *S. mansoni*. Sectors are identified by letter and are described in the text. *N*_*i *_– number of children in each sector with improved organomegaly at the 3 year follow up; *N*_*t *_– total number of children examined at the third year follow-up; *Z*-Wilcoxon Rank analysis Z score indicating whether or not there was a decrease in the degree of organ enlargement between baseline and the 3 year follow up. A negative Z value and a corresponding *p *value of <0.05 indicates a significant decrease in organ sizes within a given sector.

	*MAL splenomegaly*			*MCL splenomegaly*			*MSL hepatomegaly*			*MCL hepatomegaly*		
Sector	*N*_*i*_/*N*_*t*_	*Z*	*p*	*N*_*i*_/*N*_*t*_	*Z*	*p*	*N*_*i*_/*N*_*t*_	*Z*	*p*	*N*_*i*_/*N*_*t*_	*Z*	*p*

*A*	21/23	-3.81	<0.001	15/23	-1.20	0.234	10/23	-1.54	0.125	7/23	-0.11	0.915
*B*	9/13	-2.58	0.010	5/13	-0.42	0.673	2/12	1.39	0.164	1/12	-1.54	0.123
*C*	9/15	-2.72	0.007	5/15	-1.13	0.260	8/15	-1.22	0.222	2/13	-128	0.201
*D*	18/20	-3.78	<0.001	9/20	-2.72	0.007	12/20	-2.80	0.005	4/20	0.00	1.000
